# Social Isolation and Child Maltreatment Among Japanese Mothers: Focus on Loneliness, Social Support, and Social Cohesion

**DOI:** 10.3390/children13070897

**Published:** 2026-07-05

**Authors:** Shiqi Zhang, Takafumi Soejima, Qiting Lin

**Affiliations:** 1 Graduate School of Medicine, Kobe University, 7-10-2 Tomogaoka, Suma-ku, Kobe 654-0142, Japan; 222k053k@stu.kobe-u.ac.jp (S.Z.); linqiting@fshtcm.cn (Q.L.); 2 Foshan Hospital of Traditional Chinese Medicine, No. 6 Qinren Road, Chancheng District, Foshan 528000, China; 3 The Eighth Clinical Medical College, Guangzhou University of Chinese Medicine, Guangzhou Higher Education Mega Center, No. 232 Waihuandong Road, Panyu District, Guangzhou 510006, China

**Keywords:** social isolation, child maltreatment, social cohesion, social support, loneliness

## Abstract

**Highlights:**

**What are the main findings?**

- The association between social isolation and child maltreatment is examined across multiple ecological levels.
- Two indirect pathways link social cohesion to child maltreatment risk.
- Loneliness is identified as a key mediator in both pathways.

**What are the implications of the main findings?**

- Interventions should focus on improving neighborhood connectedness and trust.
- Professional support is also needed to reduce maternal loneliness.

**Abstract:**

**Background/Objectives**: Social isolation is a significant risk factor for child maltreatment. However, few studies have examined this relationship across multiple ecological levels, including neighborhood, family, and individual factors. This cross-sectional study operationalized social isolation using social cohesion, social support, and loneliness, and aimed to examine how their inter-relationships influence child maltreatment among Japanese mothers. **Methods**: Data were collected through an anonymous online survey of 330 Japanese mothers of children aged under six years, conducted April–May 2025. Structural equation modeling was employed in a two-step analytic approach. First, a confirmatory factor analysis was conducted to establish construct validity. Second, the hypothesized structural model was tested to examine the proposed pathways among social cohesion, social support, loneliness, parenting stress, and child maltreatment. Analyses were conducted using weighted least squares with mean and variance adjustment estimation. **Results**: Higher social cohesion was indirectly associated with reduced child maltreatment via two pathways. First, higher social cohesion was associated with lower loneliness, reduced parenting stress, and decreased child maltreatment (β = −0.063, *p* < 0.001). Second, higher social cohesion was associated with greater social support, reduced loneliness, lower parenting stress, and decreased child maltreatment (β = −0.043, *p* < 0.001). **Conclusions**: These findings highlight that enhancing neighborhood connectedness and alleviating maternal loneliness are key changes that may help to prevent child maltreatment.

## 1. Introduction

Child maltreatment is a global problem with long-term adverse consequences for health and social functioning [1,2,3]. In 2024, child guidance centers in Japan handled 223,691 child abuse and neglect cases, with biological mothers accounting for the largest proportion of perpetrators (48.2%) [4]. Social isolation is widely recognized as a significant risk factor for child maltreatment [5,6], and its effect on Japanese mothers has been identified as an important concern. Maternal social isolation in Japan is characterized by limited availability of consultation resources for child-rearing and weak social connectivity within communities [7,8,9]. Against this background, the current study examines the association between social isolation and child maltreatment among Japanese mothers. In the present study, child maltreatment was operationalized as maternal physical abuse, psychological abuse, and neglect toward children. These forms of maltreatment were selected because they are closely related to parenting behaviors and are commonly assessed in Japanese studies of child maltreatment [10,11,12]. We anticipate that the findings could provide insights for parenting support and child maltreatment prevention strategies aimed at reducing maternal social isolation and related risks.

### Literature Review

Belsky’s [5] Ecological Integrative Model of Child Maltreatment suggests that risk and protective factors operate across four nested levels: ontogenic development, which includes parents’ individual characteristics; the microsystem, representing the immediate family environment; the exosystem, comprising broader formal and informal social structures, such as parental workplaces and neighborhoods, that indirectly affect the family; and the macrosystem, representing cultural values and belief systems, which influence child maltreatment by shaping the other levels [6,13]. Belsky [5] suggested that, within the exosystem, families engaged in child maltreatment often lack access to both formal and informal support networks. Building on this ecological perspective, prior studies have measured social isolation at the individual level, focusing on subjective experiences of parental perceived social isolation [14]; at the interpersonal level, focusing on mothers’ social networks [15]; and at the community level, encompassing community integration, participation, and resources [16]. Tucker and Rodriguez [17] conceptualized maternal social isolation as the perceived lack of extra-familial social support within the exosystem and loneliness at the individual level. We build on prior research by operationalizing maternal social isolation across multiple ecological levels: loneliness in ontogenic development, social support from families and friends in the microsystem and exosystem, and social cohesion in the exosystem.

Social cohesion is a key component of collective efficacy, reflecting trust and mutual support among neighbors [18]. Prior studies have examined the associations of social cohesion and similar concepts (e.g., social capital, neighborhood connectedness) with child maltreatment, seeking ways to mitigate the risks associated with structural disadvantage such as poverty and unemployment [19,20,21,22,23]. Guterman et al. [24] found that perceived negative neighborhood processes, as measured using indicators of social disorder, informal social control, and social cohesion, indirectly predicted the risk of physical child abuse and neglect via parenting stress. A longitudinal study examining parents of children between 3 and 9 years of age demonstrated that higher levels of neighborhood collective efficacy were linked to decreased child maltreatment, mediated by reductions in parenting stress [25]. Accordingly, we proposed the following hypothesis:

**H1.**  *Social cohesion is negatively associated with child maltreatment via reduced parenting stress.*

Social support has been shown to protect against child maltreatment by buffering parenting stress [2,26,27]. Building on the work of Guterman et al. [24], Maguire-Jack and Wang [28] specifically examined the protective roles of social cohesion and social support. They found that higher social cohesion was associated with reduced child maltreatment through enhanced social support and alleviated parenting stress. We therefore proposed the following hypothesis:

**H2.**  *Social cohesion is negatively associated with parenting stress through enhanced social support and is consequently negatively correlated with child maltreatment.*

Loneliness, defined as the subjective feeling of being alone and disconnected, has been associated with various health risks [29,30]. Maternal loneliness has been identified as a significant determinant of health and well-being among perinatal women and their children. Insufficient social support has been linked to elevated maternal loneliness, resulting in depression, anxiety, and parenting stress [31,32,33,34,35]. In addition, a longitudinal UK study conducted during the COVID-19 pandemic found that neighborhood cohesion—conceptually close to social cohesion—was linked to reduced loneliness [36]. Given that parenting stress has been consistently linked to child maltreatment [24,28] and that between social cohesion and social support [28], we proposed the following hypotheses:

**H3.**  *Social cohesion is negatively associated with child maltreatment via reduced loneliness and alleviated parenting stress.*

**H4.**  *Social cohesion is negatively associated with child maltreatment via enhanced social support, reduced loneliness, and alleviated parenting stress.*

**Figure 1:**
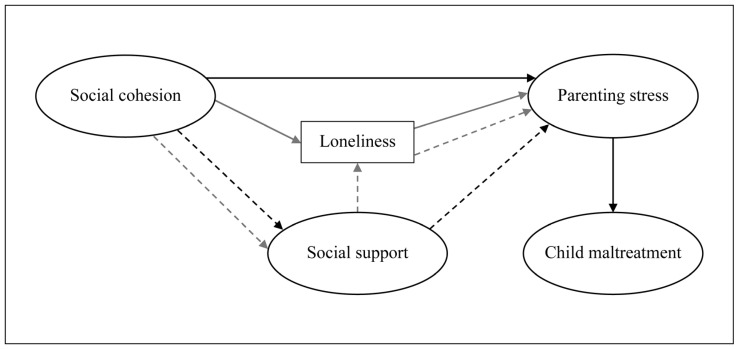
Hypothesized model of maternal social isolation and child maltreatment. Note: Solid black line = H1; dashed black line = H2; solid gray line = H3; dashed gray line = H4.

Our study extends Maguire-Jack and Wang’s [28] proposed framework by introducing loneliness as an ontogenic development factor in two novel hypothesized pathways linking social cohesion, social support, parenting stress, and child maltreatment (Figure 1). By examining how the interaction between risk factors at multiple levels affects child maltreatment, this study could provide new insights for prevention.

## 2. Materials and Methods

### 2.1. Participants and Procedure

This cross-sectional study was conducted from April to May 2025 using an anonymous online questionnaire survey commissioned from Cross Marketing, an Internet research company in Tokyo, Japan. A total of 6500 invitations were distributed to panel members. Of these, 667 respondents completed the screening survey. Only mothers aged 18–49 years who were living with and raising their youngest child under the age of six met the eligibility criteria and were invited to complete the full questionnaire. A total of 437 eligible participants completed the survey (overall response rate: 6.7%). According to the Monthly Report of Vital Statistics 2024, mothers aged 50 years or older accounted for less than 0.1% of all live births in Japan [37]. Participants who completed the survey in under five minutes (*n* = 51) or showed straight-line answering patterns across one or more items across Q8–Q11 (*n* = 56) were automatically excluded by the survey platform prior to data export, resulting in a final analytical sample of 330 mothers. This sample size was considered adequate for the structural equation model used in this study [38]. Missing data were not present in this study, as the online survey platform required participants to respond to all items before submission.

Participants were recruited through the research company’s web-based panel and gave their consent prior to completing the questionnaire. This study was approved by the Institutional Ethics Committee of the Kobe University Graduate School of Health Sciences (31 January 2025; No. 1322).

### 2.2. Measures

#### 2.2.1. Child Maltreatment

Consistent with the study’s focus on maternal maltreatment behaviors within the family context, child maltreatment was assessed based on mothers’ self-reported parenting behavior toward their youngest child, including physical abuse, neglect, and psychological abuse. These forms of maltreatment were selected because they are closely related to parenting behaviors and are commonly assessed in Japanese studies of child maltreatment [10,11,12]. Child sexual abuse was not assessed because the present study focused on parenting-related forms of maltreatment, and the Japanese-language measures adopted in this field do not typically include items assessing child sexual abuse. Items were adopted from previous studies in Japan [10,11,12] and the Parent–Child Conflict Tactics Scale [39]. Physical abuse (Cronbach’s alpha = 0.92) was assessed using six items, including hitting the child (on the buttocks, hands, or face), pinching them, and throwing objects at them. Neglect (Cronbach’s alpha = 0.69) was assessed using three items: failing to ensure the child received required food, ignoring the child when crying, and leaving them at home alone. Psychological abuse (Cronbach’s alpha = 0.67) was assessed using three items: yelling at the child, shutting them out of the house (e.g., on the balcony), and confining them in a bathroom or similar space. Mothers were asked to indicate the frequency of each behavior on a 5-point Likert scale ranging from 1 (“never”) to 5 (“always”). In the confirmatory factor analysis (CFA) results, factor loadings ranged from 0.72 to 0.82 for physical abuse, 0.50 to 0.77 for neglect, and 0.34 to 0.68 for psychological abuse. The model demonstrated excellent fit indices (goodness of fit index [GFI] = 0.983, comparative fit index [CFI] = 0.987, root mean square error of approximation [RMSEA] = 0.045, standardized root mean square residual [SRMR] = 0.082), supporting the construct validity of child maltreatment. The latent variable child maltreatment was created using the three subscale scores.

#### 2.2.2. Social Cohesion

Mothers’ isolation from the neighborhood community was assessed using the Japanese version of the Social Cohesion and Trust Scale [18,40]. The scale’s five items measure mothers’ perceptions of connectedness and trust within the community on a 5-point Likert scale ranging from 1 (“strongly disagree”) to 5 (“strongly agree”). Example items are “People in my neighborhood can be trusted” and “People in my neighborhood are willing to help their neighbors.” The scale’s Cronbach’s alpha was 0.96, and factor loadings ranged from 0.82 to 0.94. The observed items were used as indicators of the underlying social cohesion construct.

#### 2.2.3. Parenting Stress

Parenting stress was measured with the Japanese version of the Parenting Stress Index-Short Form [41,42], with all items scored on a 5-point Likert scale ranging from 1 (“strongly disagree”) to 5 (“strongly agree”). A CFA was performed to derive factor scores for parenting stress, with the four subscale scores serving as indicators: (1) parent–child dysfunctional interaction (PCDI), comprising four items (e.g., “My child rarely does things that make me happy”); (2) difficult child (DC), including five items (e.g., “Compared to other children, my child has difficulty concentrating”); (3) parental distress (PD), consisting of six items (e.g., “I am unable to enjoy activities as I did previously”); and (4) marital relationship (MR), with two items (e.g., “Since having my child, my spouse (or partner) has not provided the help and support I expected”). The Cronbach’s alpha coefficients were 0.78 for PCDI, 0.72 for DC, 0.80 for PD, and 0.60 for MR.

#### 2.2.4. Social Support

Social support was assessed using the Japanese version of the Duke Social Support Index (DSSI-J) [43], which was developed based on Koenig et al. [44]. All items were standardized to enable responses on a 5-point Likert scale, with higher scores reflecting greater social support. Based on CFA results for the three subscales, emotional support was measured using six items (e.g., “Can you rely on your family or friends in times of difficulty?”), instrumental support with four items (e.g., “Can you get help with childcare?”), and appraisal support using three items (e.g., “Can you get advice on how to handle daily life problems?”). This scale assesses support received from family or friends. Cronbach’s alpha was 0.81 for emotional support, 0.88 for instrumental support, and 0.89 for appraisal support.

#### 2.2.5. Loneliness

Loneliness was measured with the Japanese version of the University of California, Los Angeles (UCLA) Loneliness Scale (Version 3) [45,46]. This 20-item measure assesses mothers’ perceived disconnection from others on a 4-point Likert scale ranging from 1 (“never”) to 4 (“always”) (e.g., “Do you ever feel that you lack social connections with others?”). The total score, ranging from 20 to 80, was used as a continuous measure of loneliness, with higher values reflecting greater loneliness. Cronbach’s alpha was 0.92.

#### 2.2.6. Control Variables

We also included several demographic characteristics identified by prior research as related to child maltreatment: maternal age, education, marital status, maternal employment status, household income, and the youngest child’s sex [22,47,48,49,50]. Marital status (reference group = married), maternal employment status (reference group = employed), and youngest child’s sex (reference group = boy) were included as dummy variables. Maternal education level (junior school, high school, vocational school/college/university or higher) and household income (low = less than 4 million JPY, middle = 4~8 million JPY, high = more than 8 million JPY) were assessed using ordinal scales. Maternal age was treated as a continuous variable.

### 2.3. Analytic Approach

Structural equation modeling was used to examine the pathways through which social cohesion, social support, and loneliness influence parenting stress and child maltreatment. We adopted a two-step analytic approach [51]. First, the measurement model was tested using CFA to establish the validity of the latent constructs and determine whether each scale adequately reflected its intended latent variable. Second, the hypothesized structural model—based on prior research—was examined to evaluate whether the proposed causal relationships among the variables were consistent with the theoretical framework and study hypotheses. This two-step analytic approach enables clear distinction between potential deficiencies in the measurement model and those in the structural model.

All analyses were performed in R 4.5.2 using the weighted least squares mean and variance adjusted (WLSMV) estimator, which is designed for ordered categorical data, does not assume multivariate normality, and provides robust standard errors and fit statistics [22,28,52]. To examine the mediating role of loneliness in the associations between social cohesion and parenting stress and between social support and parenting stress, we tested two structural models. Model 1 excluded loneliness and assessed direct associations among the variables, while Model 2 included loneliness as a mediator in these relationships. Indirect effects were tested via bootstrapping with 2000 resamples, as this method does not impose the assumption of normality of the sampling distribution and provides more accurate confidence intervals for mediation analysis [53]. The statistical significance of path coefficients was evaluated using z-values, with a two-tailed *p* value of <0.05 as the threshold. Indirect effects were deemed significant when the 95% confidence interval (CI) excluded zero. Model fit for both the measurement and structural models was assessed using the CFI, RMSEA, and SRMR, applying conventional cutoff criteria (CFI > 0.95, RMSEA < 0.05, SRMR < 0.08) [54,55].

## 3. Results

### 3.1. Descriptive Statistics

Table 1 summarizes the descriptive statistics. The mean age of mothers was 36.52 years. Overall, 92% were married and 23% had a high school education or less. Approximately 22% reported a low household income, while 61.2% were employed, including full-time employees, part-time workers, and self-employed individuals; the other 38.8% were full-time homemakers. Regarding child maltreatment subscale scores, the physical abuse score averaged 8.81 and ranged from 6 to 27; the psychological abuse score averaged 5.03 and ranged from 3 to 13; and the neglect score averaged 4.91 and ranged from 3 to 12.

### 3.2. Model Fit

Table 2 presents the CFA results for model fit. Both the measurement model and the structural model showed adequate fit to the data. Model 1 excludes loneliness. Model 2 includes loneliness as a mediator between the predictors and parenting stress.

### 3.3. Structural Model Results

**Figure 2:**
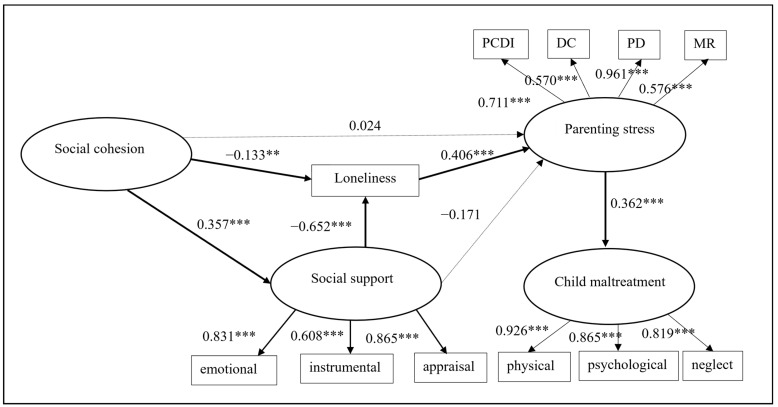
Structural Model of Maternal Social Isolation and Child Maltreatment (*N* = 330). Note: PCDI = parent–child dysfunctional interaction; DC = difficult child; PD = parental distress; MR = marital relationship. Standardized path coefficients are shown, and nonsignificant paths are denoted by dashed lines. ** *p* < 0.01, *** *p* < 0.001.

Figure 2 illustrates the associations between maternal social isolation and child maltreatment, and Table 3 presents the standardized path coefficients and their 95% CI for direct and indirect effects. Regarding the direct effects, in Model 1 (without loneliness), social cohesion was not directly associated with parenting stress (β = −0.029, *p* = 0.594), but higher social support was associated with lower parenting stress (β = −0.436, *p* < 0.001). In Model 2 (with loneliness), mothers who perceived higher social cohesion reported higher social support (β = 0.357, *p* < 0.001) and lower loneliness (β = −0.133, *p* < 0.01). Social support was negatively associated with loneliness (β = −0.652, *p* < 0.001), indicating that mothers with lower social support tended to feel lonelier. Mothers’ loneliness positively predicted parenting stress (β = 0.406, *p* < 0.001), and higher parenting stress was associated with higher child maltreatment (β = 0.362, *p* < 0.001). In Model 2, the direct association between social support and parenting stress became nonsignificant (β = −0.171, *p* = 0.072), compared with a significant association in Model 1, suggesting that this relationship was fully mediated by loneliness. The direct path from social cohesion to parenting stress remained nonsignificant in both Model 1 (β = −0.029, *p* = 0.594) and Model 2 (β = 0.024, *p* = 0.644). Both models showed excellent model fit, with Model 2 demonstrating slightly better fit than Model 1.

Social cohesion was indirectly associated with child maltreatment through two mediated pathways. First, higher social cohesion was related to lower loneliness, which in turn predicted reduced parenting stress, itself predicting decreased child maltreatment (β = −0.063, *p* < 0.001). Second, higher social cohesion was associated with greater social support, itself linked to lower loneliness, which in turn predicted lower parenting stress, which was finally related to lower child maltreatment (β = −0.043, *p* < 0.001).

Regarding control variables, mothers who were employed showed higher levels of child maltreatment compared with non-employed mothers (β = 0.172, *p* < 0.05). Mother’s age, marital status, and education, household income, and the youngest child’s sex were not significant predictors of child maltreatment. The structural model explained 16.2% of the variance in child maltreatment.

## 4. Discussion

This study examined how maternal social isolation relates to child maltreatment, with parenting stress as a mediator. It conceptualized maternal social isolation as a dynamic process, and measured it using three indicators: social cohesion, social support, and loneliness. We found that mothers who perceived lower connectedness and trust within their neighborhoods reported lower social support from family and friends, higher loneliness, greater parenting stress, and higher child maltreatment. By introducing loneliness as an ontogenic developmental factor, this study provides new insights into prevention of child maltreatment.

The present findings differ from those of Guterman et al. [24] and Barnhart and Maguire-Jack [56], who reported a direct association between social cohesion and parenting stress. By contrast, we found an indirect association through loneliness, both with and without social support. Therefore, H1 (social cohesion is negatively associated with child maltreatment via reduced parenting stress) was not supported. One possible explanation is that the level of social cohesion in Japan is relatively low compared with Western countries, which may limit its direct influence on maternal parenting stress [57]. The findings contribute to a deeper understanding of the indirect associations among social cohesion, related constructs, and child maltreatment across different cultural contexts [19,22,24,28,50,56].

Consistent with previous findings, we found a direct positive association between social cohesion and social support [28]. We also extend the framework proposed by Maguire-Jack and Wang [28] by introducing loneliness as a mediator between social support and parenting stress. Whereas their study identified a direct association between social support and parenting stress, we found an indirect pathway via loneliness, which was lower when social support was higher; in turn, parenting stress was lower. Therefore, H2 (social cohesion is negatively associated with parenting stress through enhanced social support and is consequently negatively associated with child maltreatment) was not supported. Interestingly, when loneliness was excluded from the structural model, a significant direct association between social support and parenting stress emerged. This finding suggests that loneliness mediates the relationship between social support and parenting stress. Maguire-Jack and Wang [28] recruited parents from the Supplemental Nutrition Program for Women, Infants, and Children, a federal assistance program targeting low-income mothers. They found that social support was significantly associated with lower parenting stress in economically disadvantaged families. Previous research has also shown that parenting support is more strongly associated with parenting stress among mothers with a lower (versus higher) level of education [58]. In the present study, however, ~80% of mothers had a university degree and ~80% of households were in the middle to high income range. These demographics may help explain why the direct path from social support to parenting stress was not significant.

The results are also consistent with prior findings that loneliness is associated with neighborhood cohesion, a concept closely related to social cohesion [36,59]. Whereas these previous studies focused primarily on older adults, our study observes the association among mothers aged under 50. To our knowledge, no prior study has simultaneously investigated social cohesion, loneliness, and child maltreatment among mothers. Social cohesion may alleviate maternal loneliness through mothers’ perception of trust and connectedness within the community, which enables them to access social assistance in times of need and benefit from community members’ accountability toward one another [23,60]. Consistent with Garcia et al.’s [61] findings, higher maternal loneliness was associated with greater parenting stress. Therefore, H3 (social cohesion is negatively associated with child maltreatment via reduced loneliness and alleviated parenting stress) and H4 (social cohesion is negatively associated with child maltreatment via enhanced social support, reduced loneliness, and alleviated parenting stress) were supported.

This study provides several important implications for policy and practice. It highlights that community-based interventions that take the broader social context into account may play an important role in child maltreatment prevention [62]. For instance, Strong Communities [63,64] represents a comprehensive community-based intervention that engages local volunteers and organizations to enhance children’s safety. Deployment of outreach workers can promote community engagement and facilitate the effective use of community resources, thereby potentially strengthening social cohesion and preventing child maltreatment. Our finding that social cohesion is associated with parenting stress and child maltreatment via maternal loneliness enhances understanding of the link between these variables and highlights the importance of alleviating maternal loneliness as a potential strategy for preventing child maltreatment. The findings suggest a need for interventions provided by professionals specialized in child-rearing support and child maltreatment prevention, such as social workers, clinical psychologists, and public health nurses. Such interventions should offer psychological support to enhance maternal self-esteem, and deliver social skills training to strengthen mothers’ networking and communication abilities. In addition, spouses, family members, and friends may help reduce maternal loneliness by offering emotional support, opportunities for social interaction, and practical help with childcare [65]. Mothers who experience persistent loneliness, have few people to discuss parenting with, or report high parenting stress may particularly benefit from early support, given the mediating role of loneliness identified in this study. As social cohesion was identified as a key protective factor against loneliness and child maltreatment in this study, mothers in rural or geographically isolated areas may require particular attention, since lower neighborhood density and fewer opportunities for everyday social contact may further weaken social cohesion in these settings [66]. Therefore, strengthening community ties and creating more opportunities for social participation may be crucial in these contexts. Given the central role of loneliness found in this study, ICT-based interventions—such as online parenting communities, telehealth services, and digital peer-support programs—may also help reduce maternal loneliness by facilitating social connection, particularly for mothers with limited offline networks [67,68,69].

The findings of this study should be interpreted in light of several limitations. First, its cross-sectional nature precludes causal interpretation of the association between maternal social isolation and child maltreatment. In addition, child maltreatment was operationalized as maternal physical abuse, psychological abuse, and neglect, and did not include child sexual abuse. Because this study focused on maternal parenting behaviors within the family context, the findings may not generalize to other forms of child maltreatment. Second, because child maltreatment was assessed using mothers’ self-reported parenting behaviors, social desirability bias may have led to underreporting of abusive practices. Third, social cohesion was measured based on mothers’ subjective perceptions, which may be influenced by individual biases. In particular, mothers who maltreat their children may perceive weaker social connectedness and lower levels of trust within their communities [70]. To better examine the relationship between neighborhood context and child maltreatment, future studies should incorporate objective indicators of the neighborhood environment, such as the community-level mean of social cohesion, and employ multilevel analyses to account for the hierarchical structure of individuals within neighborhoods. Fourth, although marital status was included as a control variable, the study did not assess other adverse family circumstances, such as parental intimate partner violence, mental illness, physical illness, or separation. This was because the study focused on maternal social isolation and its pathways to child maltreatment, and these variables were not included in the questionnaire to keep the survey concise, given the already low response rate. These factors may co-occur with child maltreatment and parenting stress and could have confounded the observed associations. Future studies should incorporate a broader range of family adversity variables to better isolate the unique contribution of maternal social isolation. Fifth, the relatively low response rate (6.7%; 437 of 6500 invitations) may represent a potential source of selection bias, and study participants had higher educational attainment and household income than the national average, which may limit the generalizability of the findings to the broader population. The online survey format may have further contributed to this skew, as mothers without internet access or digital literacy were unable to participate and are more likely to come from lower socioeconomic backgrounds. In addition, although the survey collected anonymous data, mothers experiencing severe economic hardship or at higher risk of maltreating their children may have been less likely to participate. Given that families from lower socioeconomic backgrounds are at heightened risk of social isolation from support systems [6], the association between social isolation and child maltreatment may have been underestimated in this study. Finally, the relationships we identified, especially the importance of parenting loneliness, need to be examined in other social and cultural contexts.

## 5. Conclusions

The present study identifies two pathways linking social cohesion to child maltreatment in Japan. In both pathways, higher social cohesion is associated with lower maternal loneliness, alleviated parenting stress, and lower child maltreatment. In the second pathway, higher social support mediates the association between higher social cohesion and lower maternal loneliness. The findings underscore that child maltreatment may be prevented through (1) community-based interventions that enhance neighborhood trust and connectedness, and (2) efforts to alleviate maternal loneliness.

## Figures and Tables

**Table 1:** Characteristics of participating mothers (*N* = 330).

	% or Mean (SD)	Range (Possible)
Mother’s age	36.52 (5.18)	20–48
Marital status		
Married	92.12%	
Unmarried/divorced/widowed	7.88%	
Education		
Junior high school	0.91%	
High school	22.12%	
Vocational school/college/university or higher	76.97%	
Employment status (employed)	61.2%	
Household income		
Low (<4 million JPY)	21.82%	
Middle (4–8 million JPY)	48.79%	
High (>8 million JPY)	29.39%	
Youngest child’s sex (boy)	47.88%	
Social cohesion	14.32 (4.92)	5–25 (5–25)
Social support		
Emotional support	20.98 (4.13)	7–30 (6–30)
Instrumental support	14.23 (3.96)	4–20 (4–20)
Appraisal support	10.85 (3.01)	3–15 (3–15)
Loneliness	45.50 (10.47)	20–77 (20–80)
Parenting stress		
Parental child dysfunctional interaction	8.34 (3.05)	4–20 (4–20)
Difficult child	13.68 (3.6)	5–25 (5–25)
Parental distress	14.77 (4.64)	6–29 (6–30)
Marital relationship	5.22 (2.17)	2–10 (2–10)
Child maltreatment		
Physical abuse	8.81 (4.17)	6–27 (6–30)
Psychological abuse	5.03 (1.82)	3–13 (3–15)
Neglect	4.91 (1.91)	3–12 (3–15)

**Table 2:** Model fit for the measurement and structural models.

	CFI	RMSEA	SRMR
Measurement model	0.999	0.037	0.046
Structural model (Model 1)	0.999	0.050	0.052
Structural model (Model 2)	0.999	0.048	0.050

**Table 3:** Direct and indirect pathway estimates from structural model testing (*N* = 330).

Path	Model 1		Model 2	
	β	95% CI	β	95% CI
Direct effects				
Mother’s age → CM Married → CM Mother’s education → CM Employed → CM Household income → CM Child male → CM Parenting stress → CM Social cohesion → parenting stress Social support → parenting stress Social cohesion → social support Social support → loneliness Social cohesion → loneliness Loneliness → parenting stress Indirect effects Social cohesion → Loneliness → parenting stress → CM Social cohesion → social support → loneliness → parenting stress → CM	−0.012 0.004 0.005 0.172 * 0.011 −0.029 0.358 *** −0.029 −0.436 *** 0.357 ***	[−0.117, 0.092] [−0.122, 0.131] [−0.128, 0.137] [0.039, 0.305] [−0.118, 0.139] [−0.143, 0.085] [0.253, 0.463] [−0.138, 0.079] [−0.552, −0.321] [0.258, 0.456]	−0.013 0.005 0.005 0.172 * 0.011 −0.029 0.362 *** 0.024 −0.171 0.357 *** −0.652 *** −0.133 ** 0.406 *** −0.063 *** −0.043 ***	[−0.117, 0.092] [−0.122, 0.131] [−0.128, 0.137] [0.039, 0.305] [−0.118, 0.139] [−0.143, 0.085] [0.257, 0.467] [−0.077, 0.125] [−0.355, 0.013] [0.258, 0.455] [−0.725, −0.579] [−0.213, −0.054] [0.229, 0.583] [−0.370, −0.138] [−0.289, −0.085]

Note: Model 1 excludes loneliness. Model 2 includes loneliness as a mediator between the predictors and parenting stress. All coefficients are standardized, except that the 95% confidence intervals (CI) for indirect effects are based on unstandardized estimates because the lavaan package in R does not provide bootstrap confidence intervals for standardized indirect effects. CM = child maltreatment. * *p* < 0.05, ** *p* < 0.01, *** *p* < 0.001.

## Data Availability

The data presented in this study are available on reasonable request from the corresponding author. The data are not publicly available due to privacy and ethical restrictions.

## Abbreviations

The following abbreviations are used in this manuscript:

CFA	Confirmatory factor analysis
CFI	Comparative fit index
RMSEA	Root mean square error of approximation
SRMR	Standardized root mean square residual
PCDI	Parent–child dysfunctional interaction
DC	Difficult child
PD	Parental distress
MR	Marital relationship
CM	Child maltreatment
